# The Killer Fly Hunger Games: Target Size and Speed Predict Decision to Pursuit

**DOI:** 10.1159/000435944

**Published:** 2015-09-24

**Authors:** Trevor J. Wardill, Katie Knowles, Laura Barlow, Gervasio Tapia, Karin Nordström, Robert M. Olberg, Paloma T. Gonzalez-Bellido

**Affiliations:** ^a^Department of Physiology, Development and Neuroscience, University of Cambridge, Cambridge, UK; ^b^Program in Sensory Physiology and Behavior, Marine Biological Laboratory, Woods Hole, Mass., USA; ^c^Department of Biological Sciences, Union College, Schenectady, N.Y., USA; ^d^Department of Neuroscience, Uppsala University, Uppsala, Sweden; ^e^Almeria, Spain

**Keywords:** Compound eye, Distance estimation, Movement detector, Object motion, Retinal velocity, Target tracking

## Abstract

Predatory animals have evolved to optimally detect their prey using exquisite sensory systems such as vision, olfaction and hearing. It may not be so surprising that vertebrates, with large central nervous systems, excel at predatory behaviors. More striking is the fact that many tiny insects, with their miniscule brains and scaled down nerve cords, are also ferocious, highly successful predators. For predation, it is important to determine whether a prey is suitable before initiating pursuit. This is paramount since pursuing a prey that is too large to capture, subdue or dispatch will generate a substantial metabolic cost (in the form of muscle output) without any chance of metabolic gain (in the form of food). In addition, during all pursuits, the predator breaks its potential camouflage and thus runs the risk of becoming prey itself. Many insects use their eyes to initially detect and subsequently pursue prey. Dragonflies, which are extremely efficient predators, therefore have huge eyes with relatively high spatial resolution that allow efficient prey size estimation before initiating pursuit. However, much smaller insects, such as killer flies, also visualize and successfully pursue prey. This is an impressive behavior since the small size of the killer fly naturally limits the neural capacity and also the spatial resolution provided by the compound eye. Despite this, we here show that killer flies efficiently pursue natural *(Drosophila melanogaster)* and artificial (beads) prey. The natural pursuits are initiated at a distance of 7.9 ± 2.9 cm, which we show is too far away to allow for distance estimation using binocular disparities. Moreover, we show that rather than estimating absolute prey size prior to launching the attack, as dragonflies do, killer flies attack with high probability when the ratio of the prey's subtended retinal velocity and retinal size is 0.37. We also show that killer flies will respond to a stimulus of an angular size that is smaller than that of the photoreceptor acceptance angle, and that the predatory response is strongly modulated by the metabolic state. Our data thus provide an exciting example of a loosely designed matched filter to *Drosophila*, but one which will still generate successful pursuits of other suitable prey.

## Introduction

Predatory animals depend on efficient prey detection for their survival and have therefore optimized their sensory systems to be particularly tuned to the movement, scent or sound of their prey [Smargiassi et al., 2012; Kane and Zamani, 2014; Kinoshita et al., 2014]. For example, barn owls localize the position of their prey using formidable hearing [Takahashi, 2010]. Accompanying this striking behavior is an enlarged and more complex auditory nucleus compared with birds that are not auditory specialists [Kubke et al., 2004]. Other predatory birds, such as kestrels, use vision to localize the prey and are subsequently equipped with a highly developed fovea that allows visualization of the prey movement from a stunning distance of 275 m [Gaffney and Hodos, 2003].

Vertebrates, which are equipped with advanced nervous systems and large brains, are clearly able to solve the task of localizing and capturing prey with amazing precision. However, insects with tiny brains and low-resolution eyes are also able to visualize the motion of small prey, as evidenced by their aerobatic and sophisticated flight behavior during predatory attacks [Olberg et al., 2000; Olberg, 2012]. Importantly, insect compound eyes have inherently poor optics [Nilsson, 1989], where the low spatial resolution can be improved by adding more lenses (resulting in larger eyes), by reducing the size of each optical ensemble (resulting in lower signal) and by creating a fovea (resulting in a localized gain in resolution, also called an acute zone) or a combination of the three. Since large eyes are heavy, increased optical resolution is associated with a substantial metabolic cost [Niven and Laughlin, 2008]. Dragonflies, which are amazingly efficient predators [Olberg et al., 2000], have huge eyes with tens of thousands ommatidia, and the best spatial resolution known among insects (down to 0.24° [Horridge, 1978]). During prey pursuit, the dragonfly aims at keeping the retinal image of the prey in the acute zone, the part of the compound eye that has the highest resolution [Olberg et al., 2000; Mischiati et al., 2015].

**Fig. 1:**
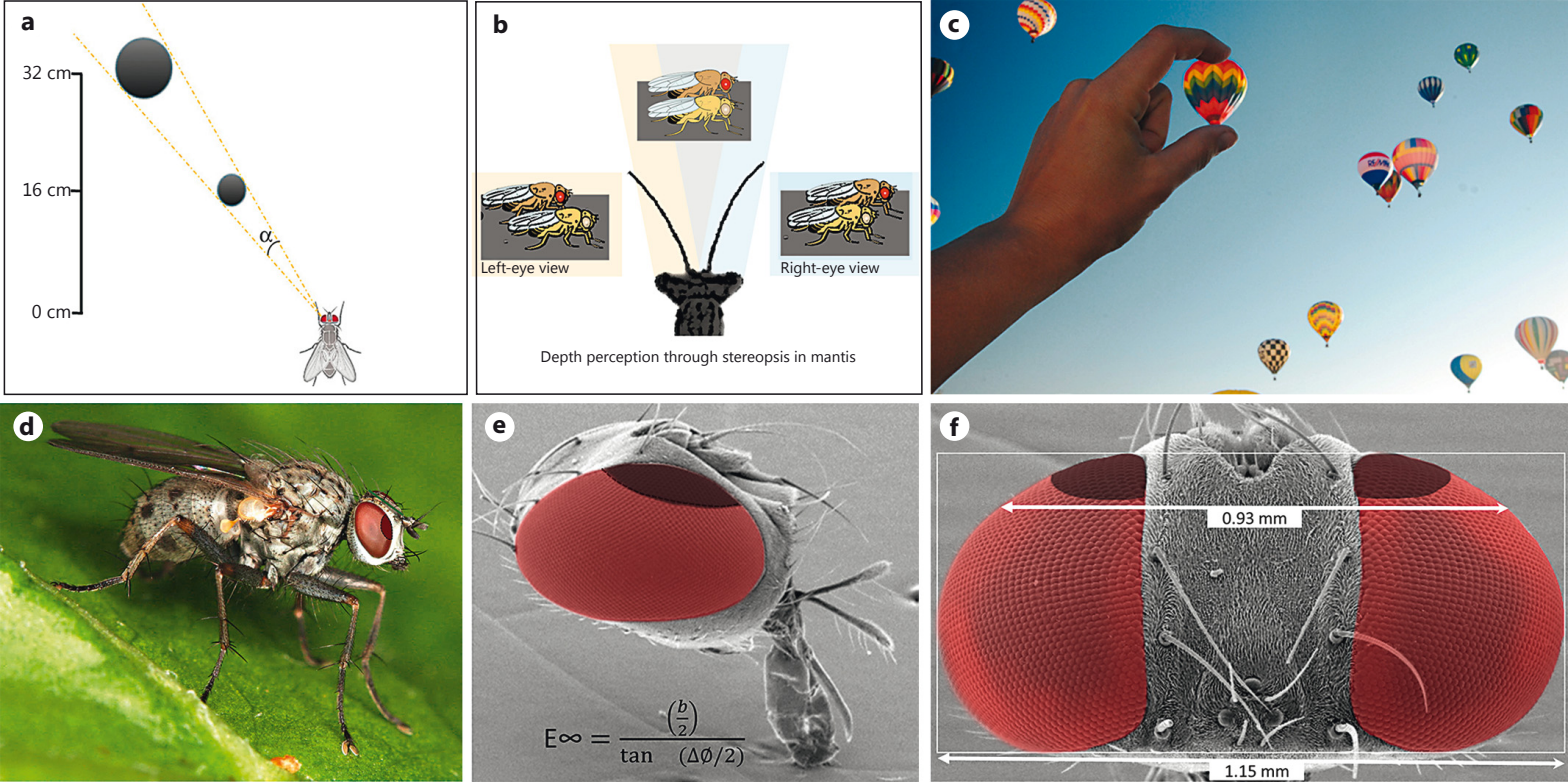
Killer flies do not possess sufficient binocularity to calculate absolute prey size prior to an attack. **a** An object that is close and small subtends the same size on the retina (α) as one that is far away and big. **b** Large insects, such as mantis, can use the retinal disparity between their eyes to calculate the true distance to an object and differentiate between both cases. **c** Without stereopsis or movements to provide motion parallax, and in the absence of other cues such as texture, discerning close-small from far-big is a difficult task. Picture credit: Sara E. Jenkins. **d-f** Killer flies have a frontal zone of increased acuity (darker red), where the cornea is flatter, but the interocular distance is not sufficient to provide stereopsis cues for their hunting range. This equation, originally proposed by Burkhardt et al. [1973], yields 2.8 cm as the maximal distance that killer flies can distinguish from infinity by binocular triangulation, where *b =* interocular separation and ΔΦ = interommatidial angle.

Before pursuing potential prey, a predator needs to know that the prey is suitable for consumption. Unless the distance to the prey is known, it is not possible to distinguish between close targets that are small and distant targets that are large, as they may subtend the same retinal angle (fig. 1a-c). This is an important problem for all predators to solve, both to minimize metabolic costs associated with pursuing unsuitable prey and to minimize the potential risk associated with breaking camouflage. Dragonflies have developed two main strategies for efficiently visualizing prey, namely perching and hovering, both aiming at rendering the background stationary, and thus making prey motion more salient [Olberg et al., 2007]. Dragonflies are very good at estimating prey size and only pursue those that are small enough to eat [Olberg et al., 2005]. Following initial visualization, dragonflies perform an impressive target pursuit mode where they predict the future path of the prey and intercept its anticipated trajectory with extremely high success rates [Olberg et al., 2000; Mischiati et al., 2015].

Distance, or depth, can be estimated using a range of techniques, with several solutions found amongst arthropods. Praying mantises, for example, use stereopsis resulting from their binocularity (fig. 1b) [Rossel, 1996], locusts use motion parallax resulting from translational movements [Sobel, 1990] and jumping spiders use the image defocus cue provided by the double retina layer present in each frontal eye [Nagata et al., 2012]. The exact mechanism employed by dragonflies to estimate prey size prior to the attack is not known, but they have been hypothesized to use motion parallax, since they perform a characteristic upward head movement before taking off to pursue prey moving above them [Miller, 1995; Olberg et al., 2005]. Such head movement provides distance information, since nearby objects will move further across the retina than far away ones. It is also possible that dragonflies use binocularity in close range, since they have a substantial portion of binocular overlap [Horridge, 1978].

Target visualization and pursuit have been studied in detail in one group of dipteran flies, namely the hoverflies. Hoverflies do not pursue prey, but vigorously chase conspecifics during territorial encounters and for potential mating [Wellington and Fitzpatrick, 1981]. Male hoverflies visualize conspecifics from a hovering stance, similarly to prey visualization by some dragonfly species. During pursuit, hoverflies calculate the future trajectory of the conspecific and set an intercepting flight [Collett and King, 1975], again like dragonflies. Importantly, however, since hoverflies only pursue conspecifics, their behavior is adapted to the assumption that the size and velocity of the target is relatively constant. This ‘hardwired’ assumption allows them to predict the distance to the target and to derive an optimal interception course without having to estimate the absolute size or distance to the target [Collett and Land, 1978]. Dragonflies and hoverflies thus use very different strategies to predict which objects are suitable to pursue.

There are much smaller dipteran species that also pursue and track targets and display exquisite flight maneuvers. Killer flies (*Coenosia attenuata*; fig. 1d) are tiny generalist predatory dipterans [Tellez Navarro and Tapia Perez, 2006], as the name implies. The interocular distance in killer flies is only 0.92 mm (fig. 1e, f) and the smallest interommatidial angle is 1.88° [Gonzalez-Bellido et al., 2011]. The maximum range for binocular distance estimation for this species of killer fly is calculated to be 2.8 cm (see equation in fig. 1e), and thus too small to provide depth information to the prey, which is typically located several centimeters away at initial visualization.

Since killer flies are generalist predators [Tellez et al., 2009], the retinal image of potential prey varies in terms of size, velocity, contrast and color. This would make a matched filter, such as a template of the prey, as exploited by hoverflies to identify conspecifics [Collett and Land, 1978], impractical. In this work, we aimed to determine the following: (1) whether killer flies compute absolute prey size prior initiating an attack and (2) what visual cues influence the decision to attack (i.e. initiate flight in pursuit of the target). We show that: (1) killer flies do not estimate absolute prey size prior to launching the attack; (2) killer flies will respond to a stimulus of an angular size that is smaller than that of the photoreceptor acceptance angle (the half width of the field of view of a photoreceptor, as measured through in vivo intracellular recordings [Gonzalez-Bellido et al., 2011]), and (3) the maximal probability of an attack being launched is when the prey's subtended size and velocity are matched to a ratio of 0.37. Finally, we show that the predatory response is strongly modulated by the metabolic state.

## Materials and Methods

### Animals

The videography recordings in the wild were carried out in two locations, either in Almeria (greenhouses, Spain) or in Massachusetts (plant nursery, USA). The laboratory tests were carried out in Cambridge (UK). The killer flies for such tests were taken from the established laboratory colony initially set up from specimens collected in Almeria (Spain).

### High-Speed Videography

For high-speed video recordings, two Photron cameras (either SA1 or SA3 models; Photron Limited, Tokyo, Japan) were used. These were positioned at a 90° angle from each other. The system was calibrated by using the MATLAB toolbox by J.Y. Bouguet's Laboratory (Caltech, http://www.vision.caltech.edu/bouguetj/calib_doc/), with code alterations. The position of the prey (or bead) and that of the predatory killer fly was digitized with custom-written MATLAB scripts. The resulting XYZ positions were fed to a fitting algorithm for trajectory reconstruction developed by Dey and Krishnaprasad [2012].

### Indoor Testing

Three testing arenas (boxes) were constructed from transparent plastic sheets (Perspex, 4 mm thick), laser cut and glued with Stix2 permanent clear tape (part No. 57084). The sizes of the boxes were: 8 × 8 × 38 cm (small), 16 × 16 × 60 cm (medium) and 32 × 32 × 38 cm (large). The lighting was provided by two sets of LED arrays (output per array 14,000 lx/1,300 foot-candles at 1 m; 4Long Model; The Light, Barcelona, Spain) placed 30 cm above the top end of the boxes. The bead was presented moving in an upwards direction, since initial tests showed that the killer flies were more likely to take off after beads in this direction than after beads moving downwards. This is in agreement with observations from Fazenda Nunes [2011].

For the bead size-velocity tests, we used at least 3 flies for each condition. We aimed to conduct 10 trials in each fly, although logistical constraints prevented this on some occasions. Nevertheless, we calculated the response from each fly as a probability of attack from the total number of trials run for each fly.

### Stimulus

The 2.14-, 5.71- and 11.9-mm diameter black beads were all made of the same material (Bead gallery, Halcraft, supplied by Michaels). The 1.33-mm bead was a silver crimping bead (Beadalon size 1), which was painted black. The 0.7-mm bead was made out of heat shrink tubing (RS Components, Corby, UK). The beads were attached to a transparent and thin fishing line (Vanish 2 LB, Berkley) using a double loop and glue. The fishing line was closed to form a loop, run along pulleys (Motion Co., Oxon, UK) which we installed in a U-shape plastic support system. The torque for the movement was provided by a 23HS-108 MK.2 stepper motor and a ST5-Q-NN DC input stepper drive Q controller (Applied Motion Products, Watsonville, Calif., USA) with a 300-watt LED power supply (LXV300; Excelsys Technologies Ltd, Rockwall, Tex., USA).

Our calibrated camera system allowed us to confirm that the velocity at which the beads were presented was a faithful representation of the chosen velocity (online suppl. fig. S1A; for all online suppl. material, see www.karger.com/doi/10.1159/000435944) and that the motor output was linear (online suppl. fig. S1B). The power of the motor translated into very fast acceleration and decelerations. Thus, the beads were already travelling at the required velocity when they entered the box.

### Hunger Trials

Ten flies formed the original test cohort, and 5 others were kept in reserve, but followed the same treatment. Each fly was tested 10 times every day, with the 1.3-mm bead presented at 340 mm/s. During the latter part of this longitudinal study, 5 flies died, likely due to starvation. To keep the study balanced for statistical purposes, the replacement flies were used in their place.

### Data Analysis and Statistics

The probability of attack for each fly is: total number of takeoffs/total number of trials. This probability was calculated for each fly tested in each condition. The representative value for each bead/velocity condition was calculated by obtaining the median probability of attack. All such calculations and plots were carried out in MATLAB (MathWorks).

The ratio of angular velocity (°/ms) to subtended size (°) was calculated with the initial assumption that killer flies measure the time taken for the position of the bead in the retina to change by 30°, and that those 30° are measured from the closest point to the bead. This simplification was used because we do not know at what point killer flies start to see the different subtended sizes. Neither do we know how long they sample each moving bead and whether this time changes according to size and velocity. This is important because the presented bead followed a straight trajectory and not a curved path. Therefore, the change in retinal position as the bead approaches the fly is not linear. Changing this value to 10 and 50° shifts the ratio to 0.3568 and 0.4044, but the significance of the actual ratio remains unchanged.

## Results

### In Natural Conditions, Killer Flies Take Off after Artificial Targets (Beads), Even When These Are Too Large to Represent Suitable Prey

**Fig. 2:**
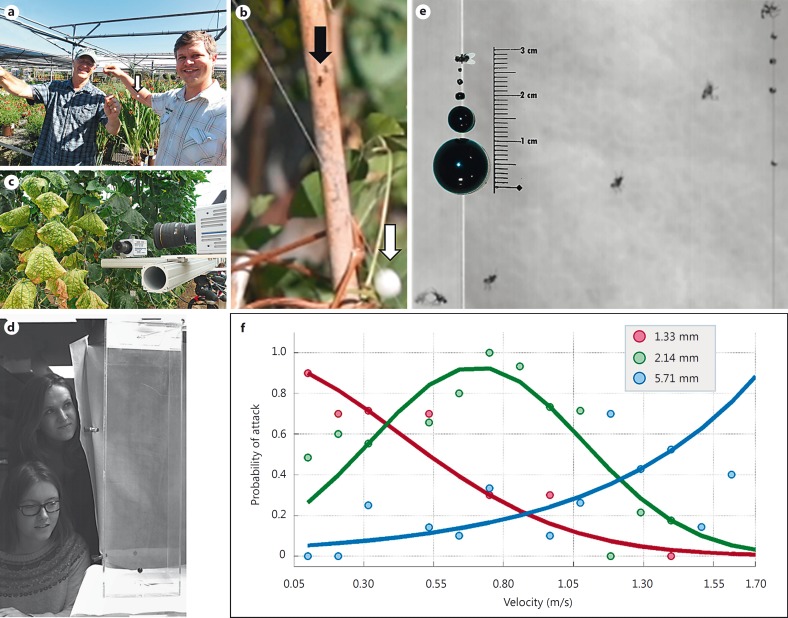
Killer flies use relative cues for assessing prey suitability. **a** Killer flies were tested in the wild (plant nursery shown in the background), with large beads presented on a string (11.92-mm bead, white arrow). **b** Killer flies attacked such beads when they were presented at high velocities. White arrow = Bead; black arrow = killer fly during attack. **c** Killer fly attacks were filmed in natural conditions (greenhouses) with high-speed video cameras (Casio F1). **d** Plastic boxes, made of suitable size to replicate the behavior observed in the wild, were used as arenas in the laboratory. **e** An overlay of snapshots along a typical killer fly hunt of the 1.33-mm bead (on the left, second bead from the top). Inset shows the five different bead sizes used in this study (killer fly included at the top for size comparison). **f** The observed probability of attack (median) shown for each velocity tested with three beads (red = 1.33 mm, green = 2.14 mm and blue = 5.71 mm). The fitted Gaussian curves reflect a tight correlation between bead size, velocity and attack probability.

A large prey located far away subtends the same visual angle on the predator's retina as a small prey close by (fig. 1a). To determine whether killer flies are able to estimate the absolute prey size prior to takeoff, we investigated pursuits initiated by killer flies in the wild (in an outdoor plant nursery and in greenhouses) by running beads of different sizes along a fishing line. The experiments took place in a naturalistic setting, where killer flies sat in their chosen perches with natural lighting and background texture (fig. 2a). This ensured that the data provided a realistic reflection of the fly's natural behavior and not a laboratory-induced artifact.

Under these naturalistic conditions, killer flies took off after plastic beads as if they were prey. Importantly, they sometimes initiated pursuit of beads that were very large (diameter of 11.92 mm; white arrow in fig. 2a, b), and thus not at all suitable as prey for the much smaller killer fly (black arrow in fig. 2b). This result suggests that killer flies do not compute absolute prey size prior to initiating an attack.

To investigate this behavior in more detail, we next quantified the natural distance at which killer flies initiate a hunt for prey. For this, we released fruit flies *(Drosophila melanogaster)* in their natural setting (greenhouses) and recorded killer fly attacks with a dual high-speed camera setup (fig. 2c). By reconstructing the position of the prey and the predator, we found that the average distance between killer fly and prey at the time of killer fly takeoff was 7.9 ± 2.9 cm (mean ± SD; n = 50).

### Suitability of Potential Prey Chosen by Killer Flies Can Be Described through a Combination of the Target's Subtended Velocity and Size

The outdoor experiments suggest that killer flies do not compute absolute prey size prior to launching an attack. To quantify prey pursuit in more controlled settings, we designed a laboratory test arena with a size that allows realistic killer fly-prey interaction. In this arena (16 × 16 × 60 cm, medium size arena; fig. 2d), we tested one fly at a time and quantified pursuits of beads of four different sizes (diameters of 0.7, 1.3, 2.14 and 5.7 mm; fig. 2e) across a range of velocities (0.10-1.72 m/s). We found that the killer flies never attacked the 0.7-mm bead, but reliably initiated a pursuit of the three larger bead sizes (fig. 2f).

By presenting the beads at different velocities, we found that the probability of attack is highly dependent on the relationship between bead velocity and bead size. For example, the 1.33-, 2.14- and 5.71-mm beads had to be presented at 0.104 (red data; fig. 2f), 0.750 (green data; fig. 2f) and 1.72 m/s (blue data; fig. 2f), respectively, to reach maximum probability of attack. Thus, the larger the bead, the faster it had to be moving to elicit a pursuit by the killer fly.

**Fig. 3:**
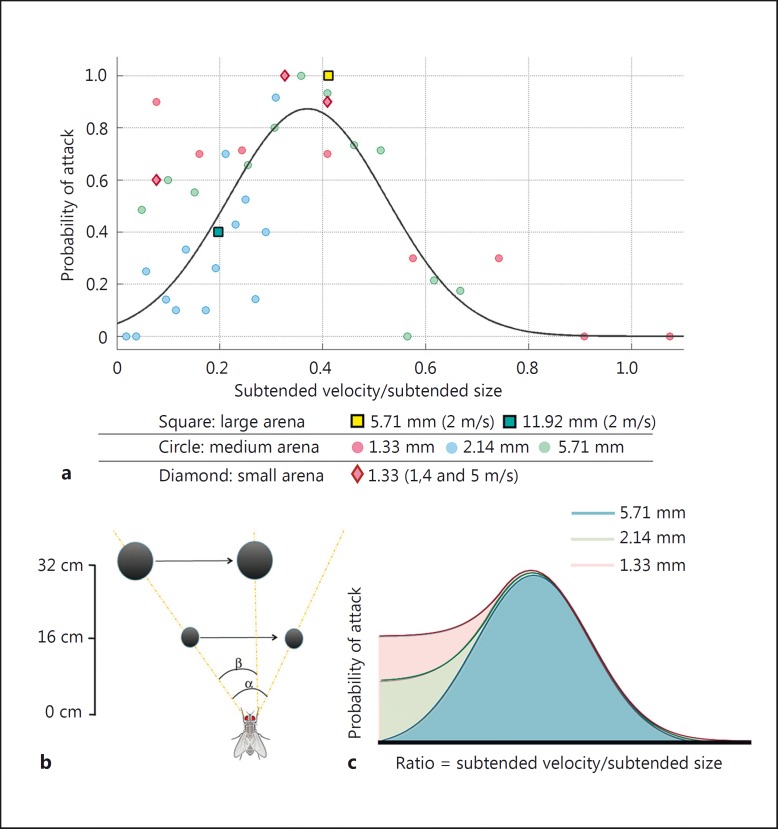
The probability of a killer fly attack is maximal when subtended prey velocity and size are proportional at a ratio = 0.37. **a** Plotting the probabilities shown in figure 2f against the ratio for each condition {ratio = [subtended velocity (°/ms)/subtended target size (°)]} aligns all data points and results in a Gaussian distribution with a peak at 0.37. Responses to the two largest beads (squares: 5.71 and 11.9 mm) tested in a larger arena follow the expected distributions. The 1.33-mm bead tested in a smaller arena also follows expected distribution at peak ratio. In addition, a high probability of attack is also seen at a ratio = 0.09 (red data). **b** The results highly suggest that killer flies can disambiguate large objects from suitable prey by linking the perceived velocity to the perceived size of the object. This is because a small object that is close will cover a wider retinal angle (α) than a large object that is far away (β) if both travel at the same velocity. **c** Stylized diagram of the results. Beads subtended a large size (5.71 mm presented at a 80-mm distance = 4.09°) will display a probability distribution explained by a Gaussian shape (blue area). Medium-size beads (2.14 mm presented at a 80-mm distance = 1.53°) will display such Gaussian distribution and in addition have a tail at the lower ratios (green area). This probability tail becomes higher as the subtended size of the object decreases (1.33 mm, presented at a 80-mm distance = 0.95°, red area).

Since absolute bead size and velocity are parameters not available to killer flies prior to the attack, a relative coordinate system must be used for this calculation. Killer flies could simply divide the subtended velocity of the prey (degrees of retina displacement over sampling time) by the subtended size of the prey. We calculated such ratio for each velocity-bead condition and plotted the probability of attack against it. The peak of a Gaussian curve fit to such results is at a ratio of 0.37 (fig. 3a). Thus, optimal parameters for suitable prey = subtended velocity/subtended bead size = ∼0.37, where subtended velocity (°/ms) = Δ retina location/Δ time and subtended bead size (°) = angular diameter at t_0_.

Such relationships provide a good fit for the majority of the data (circles; fig. 3a, same color coding as in fig. 2f). If this calculation is correct, we should be able to predict the probability of attack, even when using larger beads moving at higher velocities or at different distances. To test this, we doubled and halved the minimum fly-to-bead distance by using a large (32 × 32 cm, squares in fig. 3a) and a small (8 × 8 cm, diamonds in fig. 3a) arena, respectively. In the larger arena, we also quantified pursuits of the largest bead, which had elicited attacks in the wild settings (11.92 mm, blue square in fig. 3a).

The results confirm that this relationship is distance independent because the observed probabilities are in accordance with our predictions from the ratio described above (black line in fig. 3a shows the prediction). However, two exceptions are noted. First, in the large box, the 11.9-mm bead traveling at 4 m/s did not elicit any attacks P (expected) ∼0.9. This result could be caused by visuomuscular latencies or the reaction time, being too long for such a fast bead travelling so close to the killer fly. Second, in the small box, although the 1.33-mm bead yielded maximum probability of attack at a ratio of 0.37 (as expected), we also observed a high probability of attack when it was presented with the lowest of the tested velocities (0.1 m/s). At this velocity, this bead has a ratio of 0.7 and, thus, P (expected) = 0.1, but P (observed) = 0.7 (red data in fig. 3a). This finding is further addressed in the Discussion.

In summary, maximal attack probability is recorded and predicted when bead velocity and bead size are matched to a 0.37 ratio (fig. 3b, c). In addition, beads that subtend ≤1.53° on the retina are attacked at high rates even when presented at extremely low velocities. The smaller the subtended bead size, the lower the velocity/size ratio that will also result in a high probability of attack (fig. 3c). However, the performance of this behavior has a lower limit imposed by the minimal subtended size that can be detected by the visual system. We calculate this threshold to be between 0.5 and 0.9°, because flies never attacked the 0.7-mm bead, which at 80-mm distance subtends 0.5°, but they did pursue the 1.33-mm bead, which at the same distance subtends 0.9°.

### Takeoff Behavior Is Not a Mandatory Reflex, It Is Gated by Hunger State

The data in figure 2 show that the relationship between the angular size of the object and its angular velocity is a good predictor of killer fly attack probability. However, the results from different flies show high variability. A difference in internal drive due to hunger may explain this observation, since it is well established that hunger state is an important motivator in behavioral experiments. This makes particular sense when investigating target attacks by predators. Therefore, it is of relevance that although all flies were provided with water in our experiments, the time spent without access to food prior to testing varied between 48 and 72 h.

**Fig. 4:**
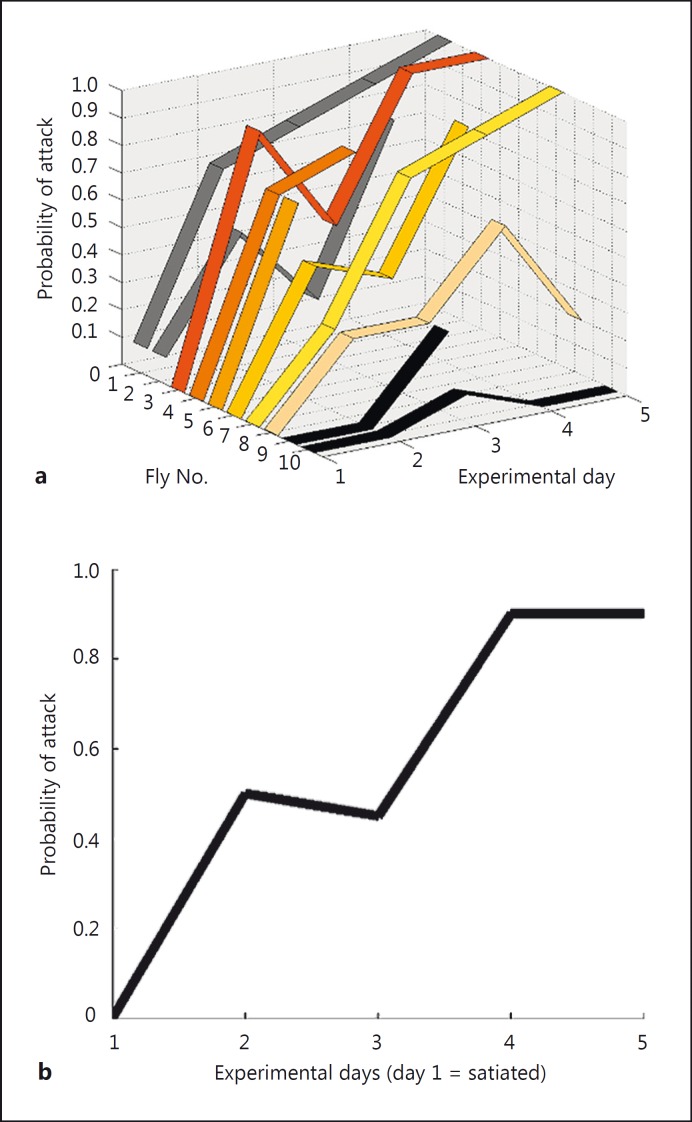
Killer fly attack response is tightly regulated by hunger state. **a** Only 2 of 10 satiated flies initiated attacks after the optimal stimulus on day 1 (flies 1 and 2, grey data), but after 24 h of isolation, all but 2 flies (fly 9 and 10, black data) initiated attack (experiment day 2). **b** Although the exact attack probability for each day was variable between flies, the median correlation between days in isolation and probability of attack is striking.

To test this hypothesis, we used 7-day-old flies that had free access to prey from the time of eclosion. After the 1st week of life, we removed access to food and recorded their probability of attack each day for 5 days. We found that when satiated, only 2 of the 10 flies attacked the bead (i.e. on experimental day 1, see flies 1 and 2, grey data in fig. 4a). After 24 h of starvation, 8 of the 10 flies attacked the bead (i.e. on experimental day 1, flies 1-8; fig. 4a). Indeed, for all flies except one (fly 10), the probability of attack increased with the number of days they were starved (fig. 4a). Across the 10 flies, we found that the probability of attack increased from 0 at day 1 to 0.9 at day 5 (median values; fig. 4b).

## Discussion

### Detection of Prey and Minimum Visual Discrimination

Before a decision to attack can be made, the prey must be detected. We found that killer flies will repeatedly and reliably attack plastic beads that subtend 0.9°, but not ones that subtend 0.5°. Thus, for killer flies, the minimum behavioral discrimination of a moving target, called the single object threshold, must be between those two values. Although this is a relatively large single object threshold for a predatory species, it is worth noting that it is much smaller than the photoreceptor acceptance angle, which is 2.8° in killer flies [Gonzalez-Bellido et al., 2011]. Hence, the image of a potential prey subtending 0.9° in the killer fly retina will only cover part of the visual field of a single photoreceptor, i.e. a target covering only 10% of the receptive field area of the photoreceptor would be detected.

Such difference between object threshold and acceptance angle has also been reported in insects that target flying conspecifics for mating purposes. In hoverflies for example, the smallest acceptance angle is 1.2°, but the object threshold is 0.32°, where its image only subtends 6% of the ommatidial sampling area [Land, 1997]. Accompanying this behavior, target-tuned neurons in the hoverfly brain respond strongly to targets that only subtend a few percent of the photoreceptor acceptance angle [Nordström et al., 2006]. This suggests that as long as the targets are moving, a very small, but continuous, drop in intensity values across neighboring facets provides a signal that permits a very low discrimination threshold. Indeed, the response of target-tuned neurons in the dragonfly brain decreases substantially if the target path is discontinuous [Dunbier et al., 2012].

It is, nevertheless, possible that the killer fly single object discrimination value was enhanced due to laboratory conditions, such as a high contrast between prey and background, plenty of light and a virtually clutter-free background. However, in our video recordings in natural conditions, we found that the minimal size subtended by a fruit fly on the retina at the time of the killer fly takeoff was ∼1.05° (mean = 2.0°), not much greater than the threshold recorded in the laboratory. Importantly, this is still much smaller than the killer fly photoreceptor acceptance angle [Gonzalez-Bellido et al., 2011], suggesting strong neural amplification of the signal [Nordström et al., 2006; Wiederman et al., 2013].

### Assessing Prey Suitability prior to Takeoff

Taken together, our results confirm that prior to launching an attack, killer flies do not compute absolute distance. This is most obvious when killer flies chase after beads that have a diameter of 11.92 mm. As figure 2b illustrates, this bead cannot possibly be a suitable target. Instead, our findings support the idea that when assessing prey suitability, killer flies make use of relative cues, and the behavior can be explained as a proportional relationship between the subtended size and subtended velocity (fig. 3).

In our laboratory study, we found that when such ratio is 0.37, the probability of attack by killer flies was close to 1. It is perhaps no coincidence that 0.37 is very close to the velocity-size ratio for a fruit fly, whose mean velocity in outdoor conditions is 1.07 m/s [Combes et al., 2012] and body length is 3 mm [Klok and Harrison, 2009]. Therefore, our results are in agreement with the notion of a matched filter to a fruit fly. Of note is the fact that the tested killer flies were reared in captivity and thus only had access to fruit flies and other killer flies as food sources. In captivity, fruit flies fly at lower velocities, circa 0.5 m/s [Ristroph et al., 2011; Combes et al., 2012] and the ratio for this value is 0.17. Our data show that killer flies will also attack targets with such a ratio (albeit with reduced probability): lower cutoff ratios for eliciting attacks are achieved by presenting targets with smaller subtended sizes (fig. 3). Taken together, the results describe the killer fly hunting strategy: an object subtending a large size must be moving fast, or it will not be a suitable prey item. However, a very small object should be suitable whether moving fast or hovering. This interpretation matches observations from a behavioral report, in which killer flies were never seen attacking prey insects that were walking (at 1 cm distance), but once the potential prey were forced into flight, killer flies attacked [Mateus, 2012].

How do killer flies obtain such optimal ratio between subtended prey velocity and size? Such a matched filter could result from a direct computation between these two parameters, but the actual process could be much simpler. A model proposed by Srinivasan and Bernard [1975] showed that the functional resolution of the retina drops when an object moves at high velocity because the voltage responses of the photoreceptors cannot reliably report the stimulus. Hence, even large objects, when presented with sufficient velocity, will produce very little modulation at the photoreceptor level and will not be detected. This explains why in our study the probability of a killer fly attack drops below 0.5 when a 1.33-mm bead is presented with a subtended velocity higher than 346°/s (estimated velocity from a bead presented at 8 cm distance, moving linearly at 0.533 m/s). Moreover, with their theoretical model, Srinivasan and Bernard [1975] also showed that as long as two consecutive objects with small (5°) separation are presented at 175°/s (a high velocity for the modeled animal), the two objects will produce the same voltage change in photoreceptors as a single object of the same size but twice the intensity. At the photoreceptor level, a large bead moving at a high velocity is equivalent to many small, consecutive objects without any separation. When the velocity is high, the subtended size of the bead will dictate the modulation of a photoreceptor signal. This explains why the 2.14-mm bead produced peak probabilities of attack at 487°/s (estimated velocity from a bead presented at 8-cm distance moving linearly at 0.75 m/s) and the 5.71 mm did so outside our tested range, but definitely higher than 1,191°/s (estimated from a bead presented at 8-cm distance moving linearly at 1.72 m/s). For the above reasons, we interpret the probability of attack observed at ratios higher than 0.37 as a failure to detect the target, and not as an actual decision to withhold the attack.

Therefore, the killer fly prey pursuit mechanism appears similar to that reported for simuliid flies, known as black flies. At the beginning of sunset, when the light availability decreases over time, the maximum distance between male and detected female also decreases [Kirschfeld and Wenk, 1976]. Although the simuliid study investigated light levels, and not velocity, the effect at the photoreceptor level is the same: a drop in photoreceptor modulation results in a lack of pursuit. Explaining why killer flies kept attacking the small beads, but not the large ones, when the size/velocity ratio is low for both is not as straight forward, but we are currently investigating different possibilities.

### The Killer Fly Attack Is Tightly Regulated by Hunger State

Our results show that although the killer fly attack can be induced reliably in most flies, it is by no means a simple reflex. Satiated flies ignored beads and probability of attack was positively correlated with the number of days in isolation (fig. 4). As a qualitative observation, when flies were satiated, they spent much of their time exploring the arena and cleaning themselves. In contrast, hungry flies quickly settled and adopted the typical hunting posture shown in figures 1d and 2e, which has been observed in 97% of the individuals seen in the wild [Mateus, 2012]. These results highlight that killer flies must monitor their metabolic needs and change their predation effort accordingly. This strategy is optimal not simply as a means to conserve energy, but also as a way of preventing being preyed upon. Killer flies are cannibalistic [Tellez Navarro and Tapia Perez, 2006], and in the absence of easier prey they will persistently hunt each other. Thus, launching an attack is linked with a probability of being killed, which must be weighed against the probability of obtaining food.

Our hunger trials further demonstrate that although the median probability of predation can be predicted when a population sample is studied, differences between individual flies persisted when age and time in isolation were accounted for (fig. 4). For example, one fly within our trials attacked the beads on one day, and only with the probability of attack = 0.1 (fly 10; fig. 4a). It is possible that this fly had fed very well before being placed in isolation, or that feeding during the larval stage provided enough energy storage. Alternatively, it is also possible that this fly was not healthy. Nevertheless, the positive correlation between hunger state and probability for predation (fig. 4b) is striking.

## Conclusion

In summary, we have shown that killer flies do not compute absolute prey size prior to attacking them and that the conditions for the highest probability of attack can be predicted with a simple proportional link between the subtended prey velocity and size. However, the fact that killer flies will also hunt very slow moving objects, as long as they subtend a very small size on the retina, calls for further investigation. The results reported here show that killer fly predation is akin to a guesstimation game: the uncertainty about prey size and location is the major limiting factor to the efficiency of the attack, which in turn limits the maximum distance to prey that results in successful capture. Whether this behavior is a result of fitting a particular niche, or a reflection of a tiny brain with relatively few neurons, remains to be elucidated.

## Supplementary Material

Supplementary data
