# Supplementary material for: The Killer Fly Hunger Games: Target Size and Speed Predict Decision to Pursuit
Source: Brain Behav Evol. 2015 Sep 24;86(1):28–37. doi: 10.1159/000435944 (PMC4612549; doi:10.1159/000435944)
Supplement: Supplementary file 1 — Supplementary data [file bbe-0086-0028-s01.pdf]

A

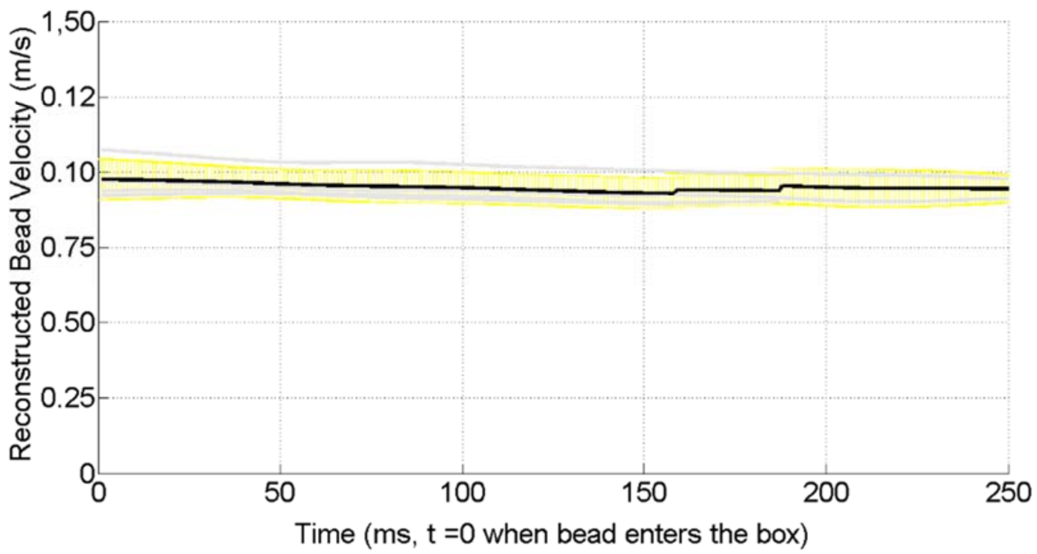

B

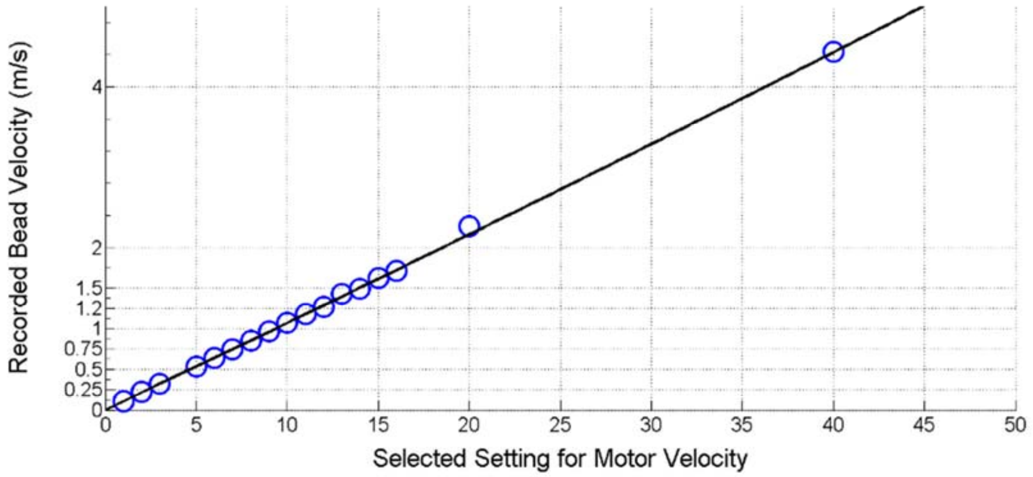

Figure S1. The motor used in this study produced a constant and predictable torque, and thus moved the presented beads with pre-set velocities. A. The actual velocity achieved by the bead was reconstructed for each velocity setting used for the motor. The example shown belongs to motor setting V9. The mean (black), SD (yellow) and grey (individual reconstructions) are shown. B. A mean velocity was computed for each motor setting by averaging the mean bead velocity recorded from the different trials. This mean is plotted against the chosen motor setting (blue circles). The agreement between chosen velocity and reconstructed velocity follows a quadratic relationship very closely.
